# Resting-state functional connectivity of the marmoset claustrum

**DOI:** 10.1162/IMAG.a.109

**Published:** 2025-08-12

**Authors:** Erin J. Holzscherer, Alessandro Zanini, Chun Yin Liu, Stefan Everling, David A. Seminowicz

**Affiliations:** Department of Medical Biophysics, Schulich School of Medicine & Dentistry, University of Western Ontario, London, ON, Canada; Department of Physiology and Pharmacology, Schulich School of Medicine & Dentistry, University of Western Ontario, London, ON, Canada

**Keywords:** claustrum, marmoset, non-human primate, resting-state functional connectivity, insula, putamen

## Abstract

The common marmoset (*Callithrix jacchus*) has been recently developed as a nonhuman primate model useful for studying behaviour, neurology, and higher-level cognitive processes considering their phylogenetic proximity to humans. Here, we investigated the resting-state functional connectivity (RSFC) of the marmoset claustrum, a small, highly connected subcortical structure. Using an open resource of 234 functional MRI scans from awake marmosets, we found claustrum connectivity to the prefrontal cortex, posterior parietal cortex, temporal cortices, cingulate cortex, sensory cortices, limbic areas, basal ganglia, and cerebellum. We also found strong functional connectivity to regions and hubs involved in marmoset resting-state networks. These findings demonstrate marmoset claustrum RSFC similar to previous human and non-human primate studies and validate the integration of marmosets into claustrum research.

## 1 Introduction

The claustrum is a thin, grey matter structure present across mammalian brains and located between the insula and putamen. It is suggested to be the most connected region relative to its size (Torgerson et al., 2015), showing widespread bilateral projections to many cortical and subcortical regions (Arrigo et al., 2017; Borra et al., 2020, 2024; Chia et al., 2020; Fernández-Miranda et al., 2008; Gamberini et al., 2017, 2021; Gattass et al., 2014; Honda et al., 2024; Jackson et al., 2020; Krimmel et al., 2019a, 2019b; Lei et al., 2025; Milardi et al., 2015; Roberts et al., 2007; Tanné-Gariépy et al., 2002; Wang et al., 2017; White et al., 2017; White & Mathur, 2018), including those involved in major networks such as the default mode network (DMN), salience network (SN), frontoparietal network (FPN), and extrinsic mode network (EMN) (Chia et al., 2020; Jackson et al., 2020; Krimmel et al., 2019b; Madden et al., 2022; Mathur, 2014; Reser et al., 2017; Rodríguez-Vidal et al., 2024; Smith et al., 2019). Recently, the network instantiation in cognitive control (NICC) model proposed that the claustrum leverages input from frontal regions to initiate, amplify, and synchronize networks during transitions between task-positive and task-negative states (Krimmel et al., 2019b; Madden et al., 2022; Reser et al., 2017). This theory is supported by claustro-cortical connections with major hubs of functional networks (Madden et al., 2022), suggesting the claustrum supports these hubs to instantiate and synchronize networks during switching. The claustrum’s extensive connectivity and proposed role in network switching have also linked it to various neurological conditions associated with disrupted network activity, including epilepsy, Parkinson’s disease, Alzheimer’s disease, and chronic pain (Arrigo et al., 2017; Ayyildiz et al., 2023; Goll et al., 2015; Kurada et al., 2019; Ntamati et al., 2023; Stewart et al., 2024; Zedde et al., 2025). However, invasive or transgenic measures used to investigate the underlying mechanisms driving these relationships are limited by the translational value of current rodent models (Chong & Gămănuţ, 2024). While rodents can provide important mechanistic insight, studies have demonstrated primate-specific evolution of cortical neurons including differences in claustral gene (Watakabe, 2017) and cell-type (Lei et al., 2025) origins and expression patterns (Pham et al., 2019; Watakabe, 2017) as well as differences in primate cortical organization and higher cognition (Magrou et al., 2024). This suggests exploring a model that is more phylogenetically, neurologically, and behaviourally similar to humans—such as non-human primates (NHPs)—can provide more extensive insight on the higher-level cognitive contributions of the claustrum.

The common marmoset (*Callithrix jacchus*) has become a popular translational model given its phylogenetic similarity to humans. Their small size and high fecundity give them an advantage over traditional Old-World primates while conserving many functional and anatomical brain network properties (Okano et al., 2012). Both cortical and subcortical subdivisions of the marmoset brain have been previously mapped (Paxinos et al., 2012; Saleem et al., 2024) and functional resting-state networks and their major hubs (Belcher et al., 2016; Ghahremani et al., 2017; Hori et al., 2020c) have been described. These networks include the DMN, SN, FPN, frontal pole network, orbitofrontal network (OFN), dorso-medial and ventral sensorimotor networks (dmSSM, vSSM), and visual networks. (Belcher et al., 2013; Burman et al., 2011; Dureux et al., 2023; Ghahremani et al., 2017; Hori et al., 2022, 2020b; Liu et al., 2019; Mitchell & Leopold, 2015). Interspecies comparisons between human and marmoset networks have revealed notable similarities in sensorimotor and visual networks, including face-, scene-, and body-specific areas (Ghahremani et al., 2017; Hori et al., 2021, 2020c; Mantini et al., 2012; Ngo et al., 2023) and motion processing networks (Cloherty et al., 2020), while human frontal networks are considered a more distributed version of the ancestral frontal networks observed in marmosets (Ghahremani et al., 2017; Hori et al., 2021). Additionally, there are existing marmoset models of many of the aforementioned disorders that potentially involve aberrant claustrum activity including Parkinson’s and Alzheimer’s diseases (Okano et al., 2012; Rizzo et al., 2021; Yun et al., 2015). Given these neurological network similarities and their recent development as a disease model, the marmoset presents a promising opportunity to investigate the network contributions and disease implications of the claustrum.

Traditionally, fMRI studies investigating the claustrum have been limited by its small size, irregular shape, and proximity to the insula and putamen. Recent work in the rodent and human have addressed this limitation by developing the small region confound correction (SRCC) which removes confounding insula and putamen signal while preserving relevant claustrum signal (Krimmel et al., 2019a, 2019b). Here, we investigated the resting-state functional connectivity of the claustrum using SRCC in awake marmosets. We found connectivity of the claustrum with widespread cortical and subcortical areas including the marmoset DMN, FPN, frontal pole network, orbitofrontal network, SN, cerebellar network, high-order visual networks, cerebellum, hippocampus, amygdala, and basal ganglia.

## 2 Methods

### 2.1 Data

Data from 31 awake marmosets were downloaded from an open access resource dataset (marmosetconnectome.org) (Schaeffer et al., 2022). As described in Schaeffer et al. (2022), all animals were acclimatized to awake MRI over many weeks of training using positive reinforcement methods and were ultimately included based on behaviour (i.e., minimal agitation). During scanning, animals were awake and monitored for comfort using an MRI-compatible camera, although physiological measures were not monitored to avoid undue stressors (Schaeffer et al., 2022). Five animals were scanned using a 9.4 Tesla (T) 31 cm horizontal bore magnet (Varian/Agilent, Yarnton, UK) at the Centre for Functional and Metabolic Mapping at Western University (UWO) (TR = 1,500 ms, TE = 15 ms, flip angle = 35°, field of view = 64 × 64 mm, matrix size = 128 × 128, voxel size = 0.5 × 0.5 × 0.5 mm, slices = 42, bandwidth = 500 kHz, GRAPPA acceleration factor: 2 (anterior–posterior)), and 26 animals were scanned using a 7T 30 cm horizontal bore magnet (Bruker BioSpin Corp, Billerica, MA, USA) at the National Institutes for Health (NIH) (TR = 2,000 ms, TE = 22.2 ms, flip angle = 70.4°, field of view = 28 × 36 mm, matrix size = 56 × 72, voxel size = 0.5 × 0.5 × 0.5 mm, slices = 38, bandwidth = 134 kHz, GRAPPA acceleration factor: 2 (left–right)). Each animal included a T2-weighted structural image and between 6 and 22 functional scans (600 volumes each (UWO), 512 volumes each (NIH)) per animal. After exclusion of 3 monkeys (missing structural scans) and 6 runs (excessive noise evaluated through visual analyses), a total of 28 monkeys with 234 runs were included in the analysis. For additional details on data acquisition and scanning parameters, see Schaeffer et al. (2022).

### 2.2 Preprocessing

All preprocessing and registration steps were performed using Analysis of Functional NeuroImages (AFNI) (Cox, 1996) and FMRIB Software Library (FSL) (Smith et al., 2004), and preprocessing code was provided through the open access resource (Schaeffer et al., 2022). The pipeline included removing the first 10 volumes of each run to account for magnetization stabilization, despiking (AFNI 3dDespike), distortion correction (FSL topup), motion correction and censoring (AFNI 3dvolreg), and bandpass removal (0.01 to 0.1, AFNI 1dBport). Phase encoding correction was performed for data from NIH animals using left–right and right–left phase encoding collected equally across scans. Preprocessing included smoothing with a 1.5 mm Gaussian kernel. However, the claustrum time series was extracted from unsmoothed data. Functional scans were registered to structural images using a transformation matrix between the mean functional image and the T2-weighted image (FSL Flirt) and were normalized using a transformation matrix between the T2 image and the NIH Marmoset Brain Mapping Atlas (V3) (Advanced Normalization Tools) (Liu et al., 2021). For detailed preprocessing and registration steps, see Schaeffer et al. (2022).

### 2.3 SRCC and seed generation

**Fig. 1.**
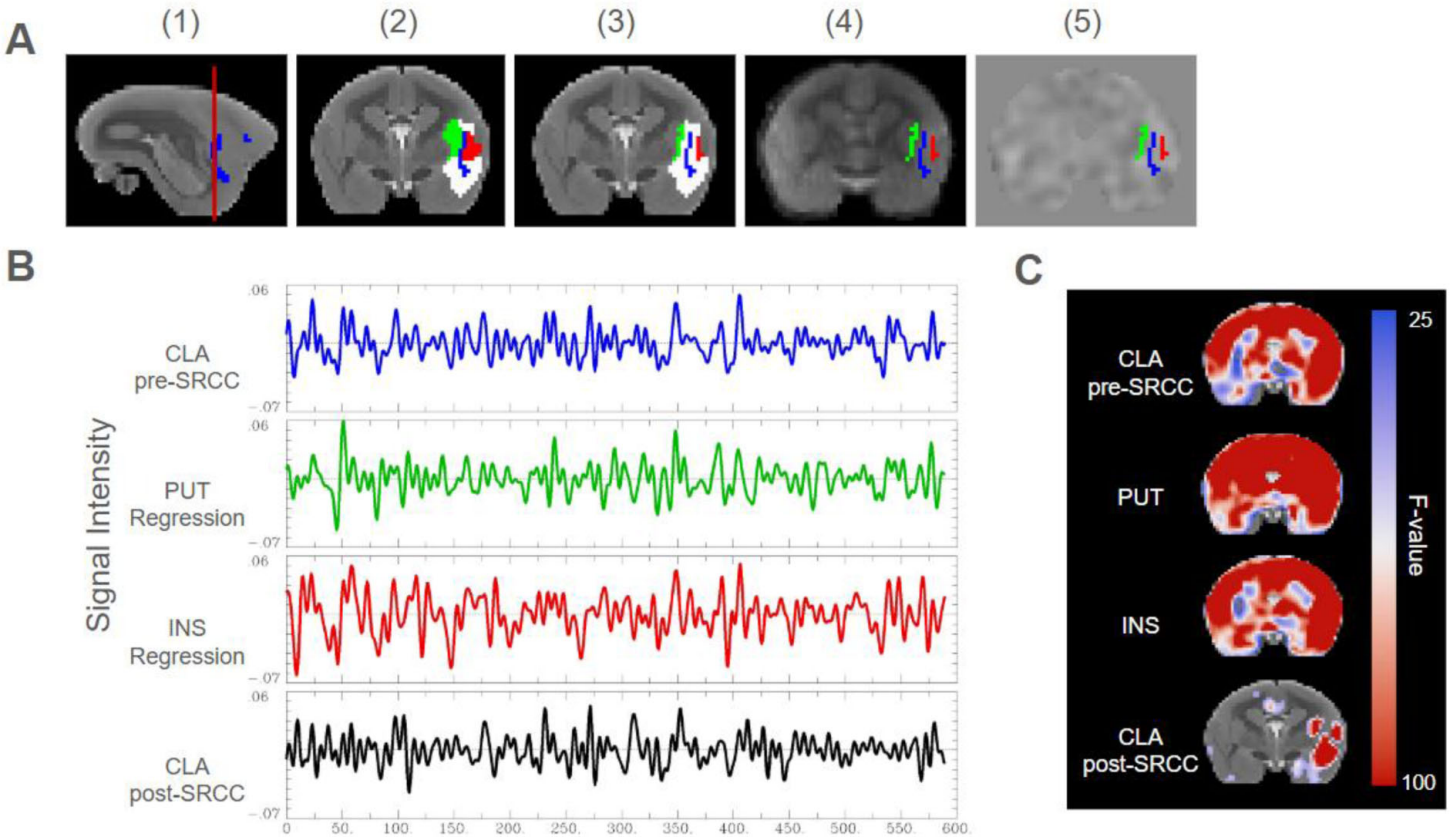
Small region confound correction (SRCC) and claustrum seed generation for the right claustrum at 0.5 mm isotropic. (A) Claustrum seed generation, (A1) slice selection on sagittal view of the marmoset template overlaid by a claustrum mask extracted from the subcortical atlas of the marmoset (Saleem et al., 2024), (A2) claustrum (blue), putamen (green), and insula masks (red), and dilated (1 mm) claustrum mask (white) used to establish overlapping regions overlayed on the marmoset template, (A3) regression regions for the putamen (green) and insula (red) created by extracting areas that overlapped between a 0.5 mm and 1 mm (white) dilated claustrum mask, (A4) claustrum (blue), putamen regression region (green), and insula regression region (red) overlayed on an individual animal’s T2-weighted image following normalization to the marmoset template, (A5) claustrum (blue), putamen regression region (green), and insula regression region (red) used to extract each time series overlayed on an individual animal’s functional scan following co-registration to a normalized T2-weighted image; (B) extracted time series for an individual animal’s normalized and co-registered functional scan for the claustrum before removal of regression regions (pre-SRCC), the putamen regression region, the insula regression region, and the claustrum following removal of regression regions (post-SRCC); (C) coronal slice of resting-state functional connectivity maps using the claustrum prior to SRCC, putamen, insula, or claustrum following SRCC as seeds. Data presented (n = 28) as significant F values (>25.987) after correction for multiple comparisons.

Small region confound correction (SRCC) was used to address partial volume effects by removing “flanking” signal from regions neighbouring the claustrum (Krimmel et al., 2019b). The claustrum was dilated by 1 voxel (0.5 mm) and 2 voxels (1 mm), and the neighbouring regions between these dilated claustrum masks (Fig. 1A) were treated as noise to be regressed out of the claustrum time series (Fig. 1B). All regions of interest (ROIs) were generated using the subcortical atlas of the marmoset (SAM) or the Paxinos atlas (Paxinos et al., 2012; Saleem et al., 2024). To determine which regions to include, the time series for each neighbouring region was correlated to the claustrum time series and compared with the time series of randomly selected spatially distant regions (Supplementary Table S1). Neighbouring regions were included in the correction if they had strong correlations to the claustrum prior to SRCC and weak correlations following SRCC when visually compared against the correlation of spatially distant regions. The correction included the putamen, and each subdivision of the insula (parainsular, dysgranular, agranular, granular, insular proisocortex) but excluded the amygdala. Claustrum seeds were then generated by extracting the left and right claustrum time series from each unsmoothened preprocessed scan and removing the time series from insula and putamen regression regions (AFNI 3dDeconvolve, 3dmaskave). To determine the efficacy of the correction, seed-to-whole-brain correlation maps were generated using the insula, putamen, and pre-SRCC claustrum as seeds. Correlation maps for each seed were inputted into a linear mixed effects model (AFNI 3dLME; Chen et al., 2013) and thresholded at the voxel level using Bonferroni correction for family-wise error (p < 0.05, critical F = 25.987) and visually compared (Fig. 1C; Supplementary Fig. S1).

### 2.4 Statistical analysis

Seed-to-whole-brain correlation analyses were performed by correlating each corrected claustrum time series to the whole brain for each individual scan (n = 234) while removing CSF as a nuisance regressor (AFNI 3dtcorr1d). White matter signal was not removed due to the risk of removing the claustrum signal (Supplementary Fig. S2). Correlation maps for each scan were then inputted into a linear mixed effects model (AFNI 3dLME; Chen et al., 2013) with no fixed effects and a random effect of subject to account for variance in the number of runs per animal. Resulting F values were thresholded at the voxel level using Bonferroni correction for family-wise error (p < 0.05, critical F = 25.987). Grey matter activation was then associated with cortical (Paxinos et al., 2012) and subcortical (Saleem et al., 2024) regions using a custom-made Matlab script which extracted the number of activated voxels, the total number of voxels, and the average F value for each cortical and subcortical ROI. The percentage of activated voxels for each labeled region was calculated and activation that encompassed less than 10% of a given region was removed from further interpretation.

### 2.5 Network classification

Significant regions were classified into networks based on Belcher et al. (2013) and Ghahremani et al. (2017) (Supplementary Table S2). The following networks were included: DMN including retrosplenial cortex, posterior cingulate cortex (23, 31, 29, 30), premotor (6DR, 6Dc, 8C), medial parietal area PGM, and posterior parietal cortex (PE, PFG, PG, LIP, MIP) (Belcher et al., 2013); dorso-medial and ventral sensorimotor networks (dmSSM/vmSSM) including primary somatosensory (1, 2, 3a–b), primary motor (4, 4c), secondary somatosensory (SP2V), cingulate cortex (23, 24), and temporoparietal regions (TPt, TPO) (Belcher et al., 2013; Ghahremani et al., 2017); higher-order visual networks including primary visual areas, V2–V6, A19, A19M, FST, TE3, A6DM (Belcher et al., 2013); SN including anterior cingulate cortex (24), anterior insula, auditory cortex, PFG, TP, and thalamus (Belcher et al., 2013; Ghahremani et al., 2017); Orbitofrontal network including A13M, A13L, orbital periallocortex, and orbital proisocortex (Belcher et al., 2013); frontal pole network including prefrontal cortex (8c, 9, A10) and A32 (Belcher et al., 2013); FPN including premotor (6DR, 8C), prefrontal (8aV, 8aD, 45, 47), medial parietal (PGM), and posterior parietal (PEC, LIP, VIP, MIP, AIP, PG) (Ghahremani et al., 2017). Cerebellar and subcortical areas were mapped and classified using the subcortical atlas of the marmoset (Saleem et al., 2024).

## 3 Results

**Fig. 2.**
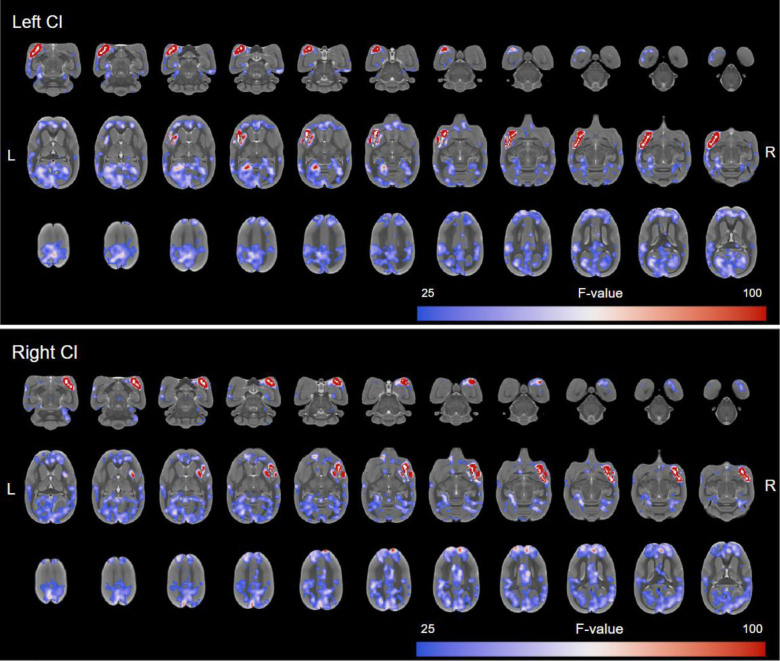
Whole-brain resting-state functional connectivity maps of the left and right claustrum following SRCC overlaid by a white claustrum mask to illustrate all the regions that are found functionally connected to the claustrum in the awake marmoset. Data presented (n = 28) as significant F values (>25.987).

For a full list and depiction of regions connected to the claustrum in the awake marmoset, see Figure 2 and Supplementary Table S3.

### 3.1 Network connectivity

#### 3.1.1. DMN

**Fig. 3.**
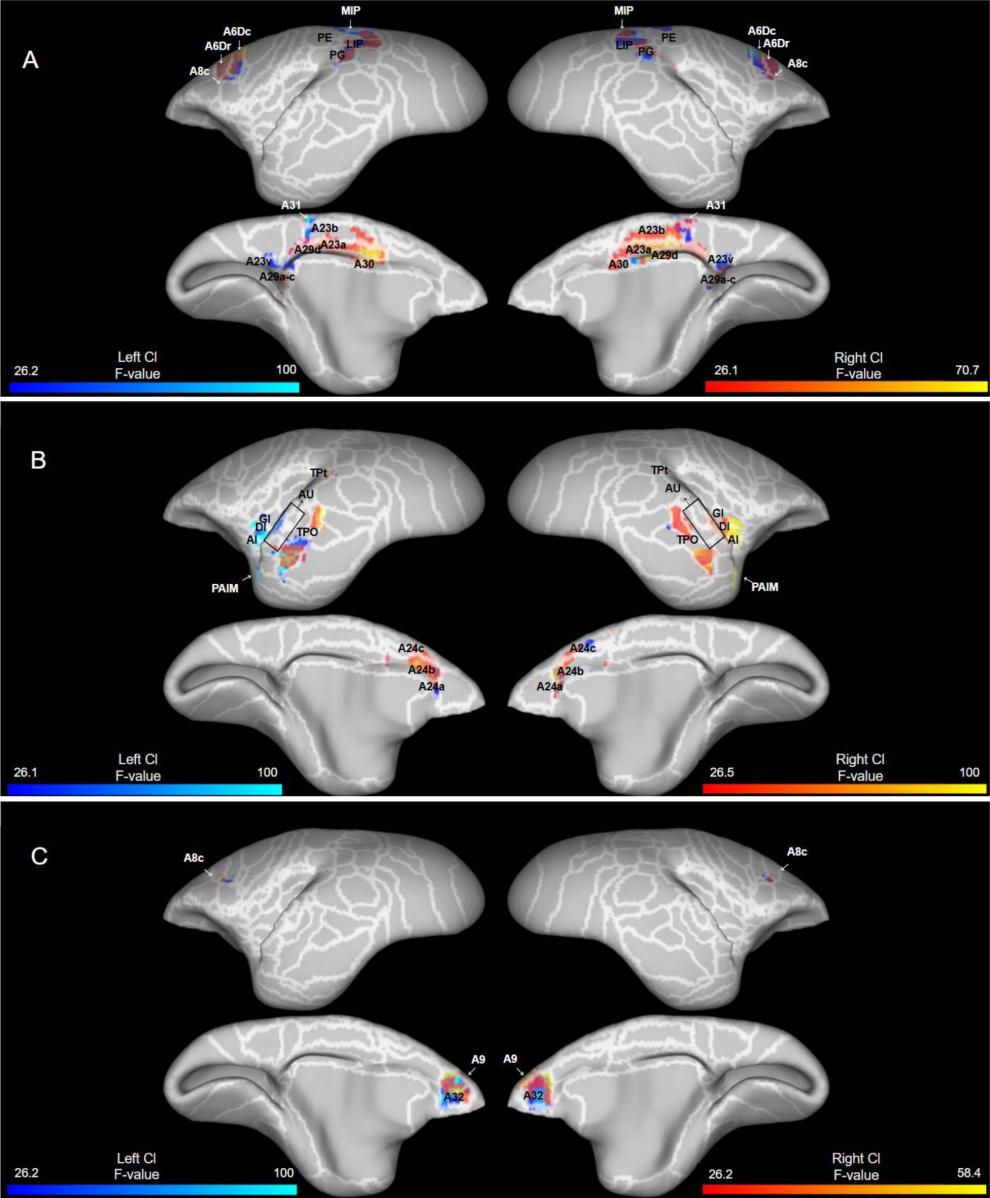

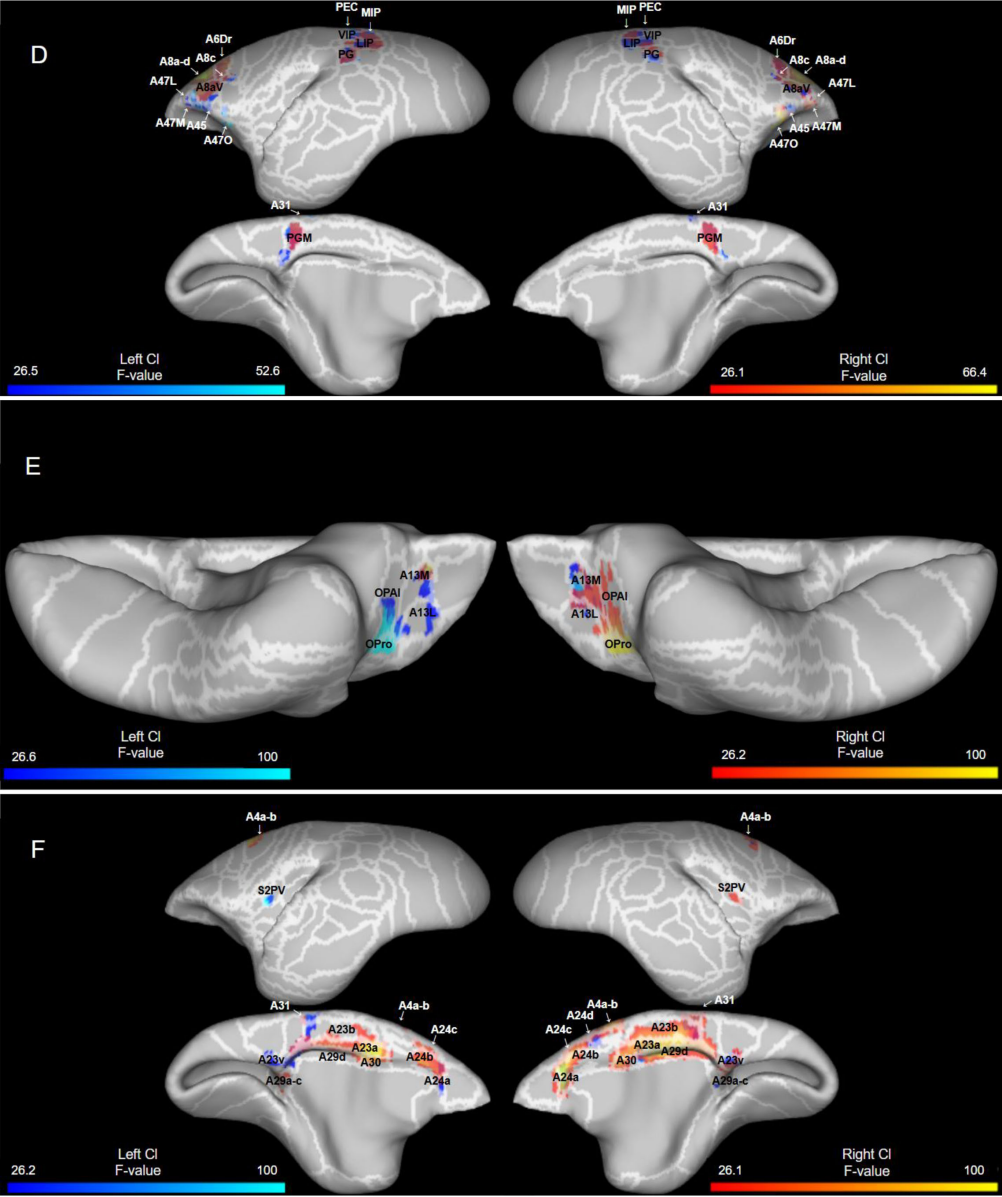

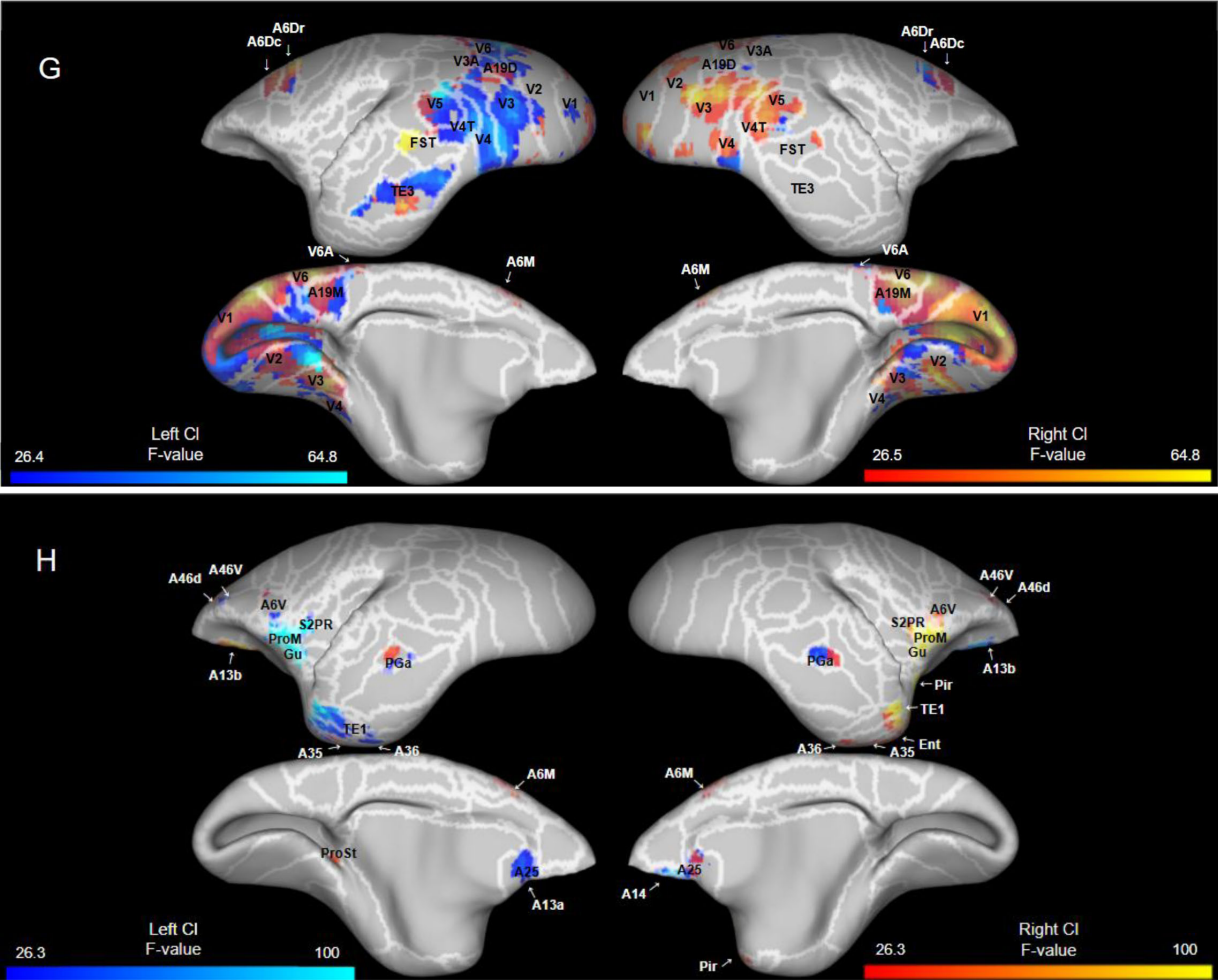
Resting-state functional connectivity of both the left and right claustrum with cortical regions: (A) Default mode network, (B) salience network, (C) frontal pole network, (D) frontoparietal network, (E) orbitofrontal network, (F) sensorimotor network, (G) visual network, (H) other cortical connectivity. Data presented (n = 28) as significant F values (>25.987) with right claustrum connectivity (red) overlaid on left claustrum connectivity (blue). White lines and cortical labels delineate the Paxinos parcellation (Paxinos et al., 2012) of the NIH marmoset brain atlas (Liu et al., 2021).

Both the left and right claustrum seeds showed strong connectivity with regions associated with the marmoset default mode network (Fig. 3A) including bilateral connectivity with the posterior parietal cortex (LIP, MIP, PG), premotor cortex (6Dr, 6Dc, 8C), and posterior cingulate cortex (23, 30). The left claustrum showed bilateral connectivity with area 31, and contralateral connectivity to PE while the right claustrum showed only ipsilateral connectivity with area 31 and no significant connectivity with PE. The right claustrum reported bilateral connectivity with area 29a–d, while the left claustrum only showed contralateral connectivity in 29d.

#### 3.1.2 SN

Both the left and right claustrum seeds showed strong connectivity with regions associated with the marmoset salience network (Fig. 3B) including bilateral connectivity with the anterior cingulate cortex (ACC, particularly in 24a–b) and ipsilateral connectivity with insular regions. The right claustrum showed bilateral connectivity with the auditory cortex, TP, and 24c while the left claustrum showed only ipsilateral connectivity with auditory cortex, and TP, and contralateral connectivity to 24d.

#### 3.1.3 Frontal Pole

Both the left and right claustrum seeds showed strong connectivity with regions associated with the marmoset frontal pole network (Fig. 3C) including bilateral connectivity with the dlPFC (8c, 9) and vACC (32).

#### 3.1.4 FPN

Both the left and right claustrum seeds showed strong connectivity with regions associated with the marmoset FPN (Fig. 3D) including bilateral connectivity with the posterior parietal cortex (LIP, MIP, PG, PGM), premotor cortex (6Dr, 8C), vlPFC (8aV, 8aD, 47), and ipsilateral connectivity with area 45. The left claustrum showed bilateral connectivity with PEc and VIP while the right claustrum showed only contralateral connectivity with VIP.

#### 3.1.5 OFN

Both the left and right claustrum seeds showed strong connectivity with regions associated with the marmoset orbitofrontal network (Fig. 3E) including bilateral connectivity with area 13 M and area 11. The left claustrum showed contralateral connectivity with area 13 L while the right claustrum only showed ipsilateral connectivity. Both the left and right claustrum also showed ipsilateral connectivity to the orbital periallocortex and orbital proisocortex.

#### 3.1.6 SSM

Both the left and right claustrum seeds showed strong connectivity with regions associated with the marmoset dmSSM (Fig. 3F) including bilateral connectivity with cingulate cortex (areas 23, 24, and 30). The left claustrum showed bilateral connectivity with area 31 while the right claustrum only showed ipsilateral connectivity. Somatosensory regions (3, 1/2, S2I, S2E) involved in the dmSSM did not show significant connectivity with either claustrum seed while primary motor cortex (A4ab) showed bilateral connectivity with the right claustrum and contralateral connectivity with the left claustrum. For the vSSM, only the secondary somatosensory cortex (SP2V) showed significant ipsilateral connectivity with the left and right claustrum.

#### 3.1.7 Higher order visual

Both the left and right claustrum seeds showed strong connectivity with regions associated with the marmoset higher-order visual networks (Fig. 3G) including bilateral connectivity with visual cortex (V1, V2, V3, V3A, V4, V5, V6, V6A), area 19, and area 23 V. Only the right claustrum showed bilateral connectivity with FST while both the left and right claustrum showed connectivity with only the left inferior temporal cortex (TE3).

#### 3.1.8 Other cortical (non-network) connectivity

Both the left and right claustrum showed strong connectivity with other cortical regions that are not defined by marmoset RSNs (Fig. 3H) including bilateral connectivity with dlPFC (46), mPFC (14), supplementary motor area (6 M), OFC (13a–b), occipitoparietal transitional area, PGa/IPa, and vACC (25). Both the left and right claustrum showed ipsilateral connectivity with gustatory cortex, A6Va–b, secondary somatosensory cortex (S2PR), ventral temporal cortex (35, 36), and proisocortical motor region. The lateral/inferior temporal cortex (TE1) and prostriate area showed mostly ipsilateral connectivity with both seeds while only the left seed had contralateral connectivity with the prostriate area. The entorhinal cortex and piriform cortex showed only ipsilateral connectivity with the right claustrum.

### 3.2. Subcortical connectivity

**Fig. 4.**
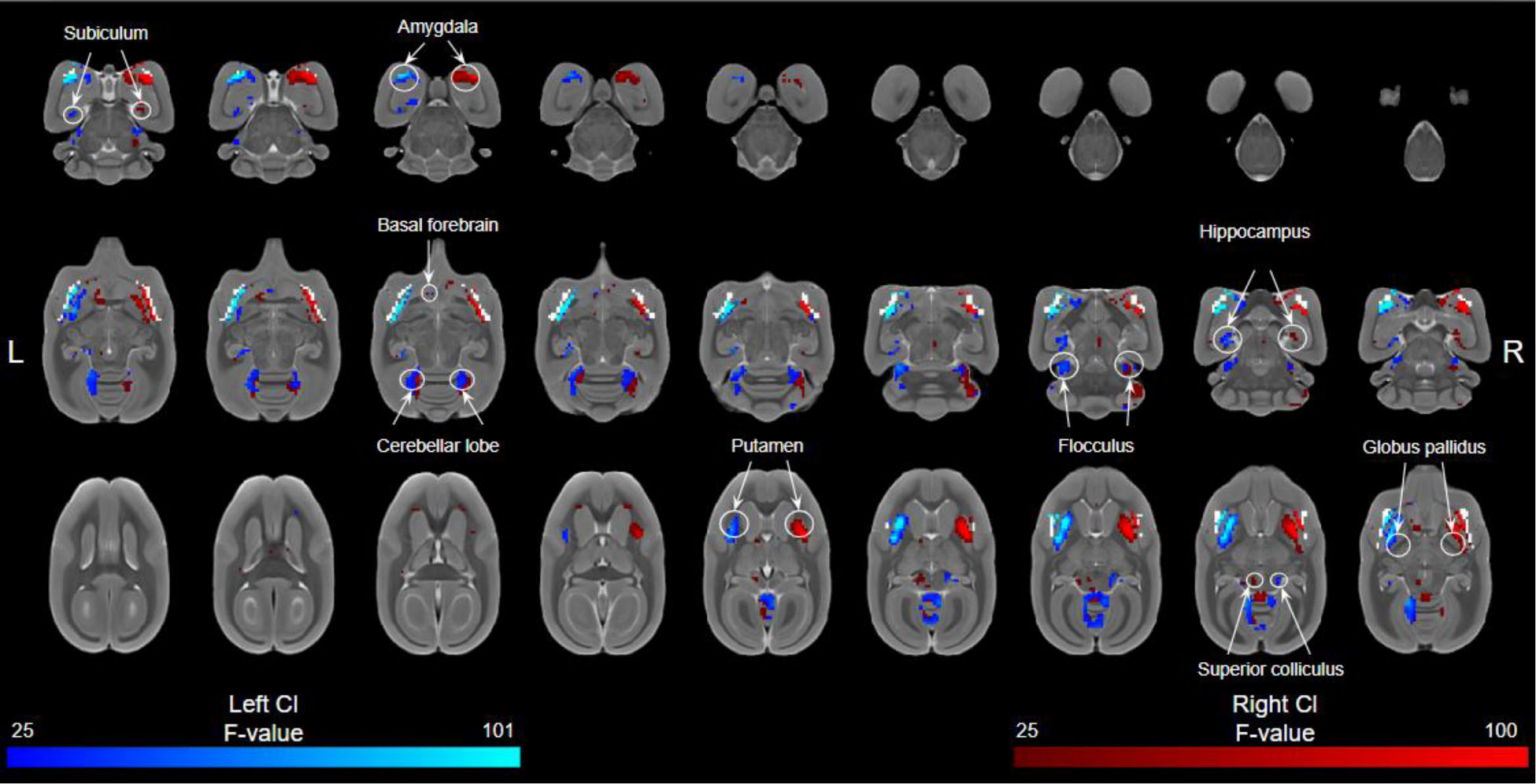
Resting-state functional connectivity of both the left and right claustrum with subcortical regions (RCl > LCl) overlaid by a white claustrum mask. Data presented (n = 28) as significant F values (>25.987) with blue representing left claustrum connectivity and red representing right claustrum connectivity.

Both the left and right claustrum showed strong ipsilateral connectivity with subcortical regions (Fig. 4) including the basal ganglia (globus pallidus, putamen), amygdala, anterior cortical nucleus, hippocampus (CA4, dentate gyrus), and olfactory tract. Both the left and right claustrum showed bilateral connectivity with the basal forebrain and subicular regions and contralateral connectivity with the superior colliculus. The right claustrum showed contralateral connectivity with the nucleus accumbens while the left claustrum showed ipsilateral connectivity with the posterior cortical nucleus and olfactory tubercle.

Both the left and right claustrum seeds showed strong connectivity with the cerebellum including bilateral connectivity with cerebellar lobule IV and anterior part of the simple lobule. The left claustrum showed bilateral connectivity with cerebellar lobule V while the right claustrum showed only contralateral connectivity. Both seeds showed ipsilateral connectivity with the flocculus while the left claustrum showed ipsilateral connectivity with cerebellar lobule III and the right claustrum showed ipsilateral connectivity with crus I of the ansiform lobule.

## 4 Discussion

The purpose of this study was to explore the resting-state functional connectivity of the claustrum in the awake marmoset. Previous work has demonstrated a significant dampening effect of isoflurane on rsfMRI which can limit exploration of network connectivity with the claustrum in animal models (Hori et al., 2020a; Smith et al., 2017). Additionally, the small size, irregular location, and proximity to neighbouring regions further limit its ability to be analyzed through fMRI due to partial volume effects (Krimmel et al., 2019b; Madden et al., 2022; Reser et al., 2017). Here, we addressed these previous limitations by employing the small region confound correction, a technique used to remove confounding signals from neighbouring regions on awake marmosets (Krimmel et al., 2019b). Using 234 awake marmoset functional scans, we found left and right claustrum connectivity with prefrontal cortex, cingulate cortices, posterior parietal cortex, temporal cortex, primary sensory cortices (visual, auditory, motor), secondary sensory cortices (visual, motor, somatosensory), amygdala, basal ganglia, hippocampus, and cerebellum. We found connectivity to many major regions and hubs of marmoset resting-state networks including both left and right claustrum connectivity to hubs of the DMN (23b/32), salience network (24), and visual networks (V1/V2/19M/V6DM), as well as to major regions of frontal networks such as FPN (dlPFC) and orbitofrontal network (11, 13).

Considering previous challenges in isolating claustrum signal from neighbouring regions, we used a novel correction (SRCC) developed in rodents and humans to remove confounding insula and putamen signal. Using this method, we were able to show functional connectivity maps that have distinct patterns from insula and putamen functional connectivity maps. For example, previous anatomical work differentiating ACC input (A24, A32) between the two regions has shown that A24d has sparse connections with the marmoset claustrum yet extensive connections with the insula (Reser et al., 2017). Here, we find functional connectivity with the claustrum and ACC is concentrated mostly in A24a–c and has few connections with A24d, while functional connectivity patterns of the insula and claustrum pre-SRCC show connectivity across all regions of the ACC. Additionally, functional connectivity patterns of the claustrum pre-SRCC are similar to both putamen and insula functional connectivity patterns, while patterns post-SRCC are relatively distinct. Taken together, these findings support the efficacy of SRCC on isolating the claustrum by removing the majority of confounding insula and putamen signals in the marmoset.

Generally, our findings support the claustrum’s widespread connections across many cortical and subcortical regions as seen through previous non-human primate anatomical work. This includes regions such as prefrontal cortex, specifically A8aD and A45 (Roberts et al., 2007), anterior cingulate cortex (A24b–c, A32, A32V) (Reser et al., 2017), and hippocampus (presubiculum, parasubiculum, subiculum) (Honda et al., 2024) in the marmoset and prefrontal cortex (Lei et al., 2025; Pearson et al., 1982; Tanné-Gariépy et al., 2002), frontal-motor areas (Lei et al., 2025; Tanné-Gariépy et al., 2002), parietal cortex, specifically parietal-sensory (Lei et al., 2025), and areas PEc, PGM, MIP (Gamberini et al., 2017, 2021), ventral temporal cortex (Gattass et al., 2014; Lei et al., 2025; Pearson et al., 1982), visual areas, (Gattass et al., 2014; Lei et al., 2025; Pearson et al., 1982), putamen (Borra et al., 2020; Lei et al., 2025), and amygdala (Borra et al., 2024; Lei et al., 2025) in the macaque. However, we did not find functional connectivity with thalamic nuclei which has previously shown anatomical connections in the macaque (Borra et al., 2024; Erickson et al., 2004; Lei et al., 2025) or area 10 which has anatomical connections in the marmoset (Burman et al., 2011).

In line with previous work in humans, we also found functional connectivity to many regions and hubs of resting-state networks. Specifically, human claustrum FC studies show connectivity with specific nodes of the salience network (Rodríguez-Vidal et al., 2024), default mode network (Krimmel et al., 2019a; Rodríguez-Vidal et al., 2024), FPN (Rodríguez-Vidal et al., 2024), sensorimotor network (Rodríguez-Vidal et al., 2024), and extrinsic mode network (Krimmel et al., 2019a), while here nodes of comparable marmoset networks such as the salience network, sensorimotor network, default mode network, frontal pole network, and frontoparietal network all show bilateral connectivity with both the left and right claustrum. Also consistent with human findings, we observed asymmetry in the functional connectivity patterns of the left and right claustrum. Previous human studies have found differential responses of the left and right claustrum to pain and cognitive tasks (Stewart et al., 2024) as well as general differences in RSFC, including to major regions of the salience network, default mode network, and frontoparietal network (Rodríguez-Vidal et al., 2024). Our results support this claustral asymmetry in the marmoset given differences in the laterality of the left and right claustrum to the default mode network (inferior parietal, posterior cingulate), salience network (dACC), frontoparietal network (posterior parietal), other cortical areas (entorhinal cortex, piriform cortex), and subcortical areas (nucleus accumbens, olfactory tubercle, posterior cortical nucleus, cerebellum). Although the role of this claustrum laterality in humans is currently unknown, future studies can use this shared feature of claustrum connectivity to investigate its role using more invasive anatomical methods.

Functionally, the claustrum has been recently theorized to instantiate and synchronize functional networks as supported by this connectivity to frontal and posterior hubs of task-positive and task-negative networks (Madden et al., 2022). In line with previous work in both non-human primates and humans (Gattass et al., 2014; Jackson et al., 2020; Krimmel et al., 2019b; Lei et al., 2025; Madden et al., 2022; Pearson et al., 1982; Reser et al., 2017; Rodríguez-Vidal et al., 2024; Smith et al., 2019; Tanné-Gariépy et al., 2002), we found the marmoset claustrum showed functional connectivity with frontal and posterior hubs of marmoset-specific task-positive networks (ACC, dlPFC, PPC) and task-negative networks (dlPFC, A23b, A31). This connectivity to multiple frontal regions is suggested to support the theory of claustrum network modulation given similar topographic regions of the claustrum show projections to frontal areas that are involved in task-positive networks and those that are not. Specifically, Reser et al. (2014) propose that claustrum projections that provide input to both frontal regions of the salience network (A12) and other cortical areas (A9 and A10) in the capuchin may act to desynchronize regions of task-negative networks and instantiate task-positive networks following external cues. These shared claustral connections are further validated through similar patterns of connectional architecture found in the macaque where the same claustral zones project to multiple functional areas (Borra et al., 2024). Our findings support these patterns in the marmoset given functional connectivity patterns exist between similar frontal regions of both functional networks (A47, old A12) and non-network frontal areas (A9), as well as across many different major hubs of resting-state networks. However, future anatomical studies are needed to determine whether these connections originate from similar zones or topographic regions. We also found significant connectivity across several regions in the posterior cingulate and visual cortices which are believed to receive context-dependent signals from these frontal regions through the claustrum (Madden et al., 2022), further supporting the possible role of the marmoset claustrum in cortical network regulation. These findings lay precedent for future studies to investigate theories of claustral network modulation as well as its involvement in aberrant network disorders such as Parkinson’s, Alzheimer’s, and chronic pain through newly developed marmoset disease models.

The marmoset claustrum also showed significant connectivity with subcortical network regions such as the amygdala, hippocampus, cerebellum, and putamen. These findings are consistent with previous primate claustrum anatomical connectivity found across the hippocampus, amygdala, and striatum (Borra et al., 2020, 2024; Gattass et al., 2014; Honda et al., 2024; Lei et al., 2025) as well as human claustro-connectivity with the hippocampus, amygdala, and basal ganglia (Fernández-Miranda et al., 2008; Krimmel et al., 2019b; Milardi et al., 2015; Rodríguez-Vidal et al., 2024). Interestingly, consistent with human findings, we found functional connectivity between the marmoset claustrum and the cerebellum (Rodríguez-Vidal et al., 2024), which is absent in rodents. In humans, this cerebellar connection has a potential role in socializing (Rodríguez-Vidal et al., 2024). In this study, this functional connectivity of the marmoset claustrum with the cerebellum, as well as with other regions critical for marmoset social processing (8b, 9) (Hori et al., 2021), may support the involvement of the claustrum in socializing processes even in this New-World non-human primate. Additionally, claustrum connectivity to the presubiculum, parasubiculum, and entorhinal cortex is present both anatomically (Honda et al., 2024) and functionally in marmosets but absent in rodents. This connection is suggested to integrate memory formation with higher level cognition such as attention or self-awareness (Honda et al., 2024). These findings highlight the advantages of using the marmoset for claustrum research, as their similarities in social systems, higher cognition, and neural underpinnings (Miller et al., 2016) may allow for greater insight into the claustrum’s role in social network processing and memory integration.

### 4.1 Limitations

While SRCC is an effective method of removing confounding insula and putamen signals, it is a relatively conservative approach and may remove relevant claustrum signals (Krimmel et al., 2019b). This possible loss of claustrum signal may explain the lack of connectivity with the thalamus, hypothalamus, and area 10 seen in past research in marmosets, macaques, and humans (Burman et al., 2011; Honda et al., 2024; Lei et al., 2025; Madden et al., 2022) given the selective nature of claustrum projections (Lei et al., 2025) and considering connectivity to thalamic nuclei disappeared following the regression of the putamen. Specifically, considering the well-established topographic organization of the claustrum (Borra et al., 2024; Lei et al., 2025; Reser et al., 2014), certain projections to thalamic nuclei or frontal regions may have been removed following the removal of neighbouring insula and putamen regions. Future studies with increased resolution (e.g., 0.2 mm isotropic) can address this limitation by reducing the amount of signal included in the regression to avoid removing relevant claustrum projections. Additionally, because of the small, irregular size of the claustrum, data included in the time series were not smoothed and white matter was not removed during correlations, which may have introduced excess noise during seed generation and analyses. However, considering the large sample size of the data set and the consistency of our findings with the literature, these methods likely did not have a notable effect on the cerebral connectivity pattern of the marmoset claustrum.

### 4.2 Conclusion

Overall, our data demonstrate widespread claustrum connectivity with cortical and subcortical regions consistent with previous claustrum research. Given the connectivity across frontal and posterior hubs of major resting-state networks, our data also suggest similar theories of claustrum function including network instantiation and task switching. However, considering marmosets and humans brains are not exact homologues and have inherent differences in cortical structure and function, caution should be used when interpreting the functional implications of these results (Kaas, 2020; Ngo et al., 2023). Future studies should investigate direct interspecies comparisons of functional connectivity using task-based methods such as movie-driven fMRI (Hori et al., 2021). Taken together, our findings act as a proof of principle by demonstrating that claustrum connectivity can be explored through a novel, phylogenetically similar animal model to facilitate future functional or disease explorations of the claustrum.

## Supplementary Material

Supplementary Material

## Data Availability

All raw data, preprocessing, and registration code are described in Schaeffer et al. (2022) and available through the Marmoset Functional Brain Connectivity Resource (https://www.marmosetbrainconnectome.org/). SRCC and custom-made codes are available for download (https://osf.io/mga2v/?view_only=7aece5454cec49b2ae4956ad74959d4d).
